# 

**Published:** 1912-08-17

**Authors:** 


### A DOCTOR'S WILL.

Dr. Thomas Gilbert Emerson, of Woodford Green, Essex, lately Medical Officer of Health to the Wantage Urban District Council, who died on June 17, left estate valued at £21,957 gross, with net personalty £20,939.

### BUREAU OF INFORMATION.

### Rules for Correspondents.

1. Every letter, on whatever subject, **must be accompanied by the coupon** to be cut from the back cover (inside page) of The Hospital, current issue, and must contain the name and address of the correspondent with pseudonym for publication if desired.
2. Replies by post cannot be given, save under exceptional circumstances at the Editor's discretion.
3. Letters from Approved Homes in reply to special needs published in the Bureau should state terms and full particulars, and be sent prepaid under cover to the Editor of the Bureau with coupon and name enclosed for identification.
4. Proprietors of Homes which have not yet been entered on the List of Approved Homes, but have spare accommodation likely to suit special needs, are invited to write for an application form for registration. The fee for registration is 5s.
5. Special needs of every description relating to those who are sick or in difficulties are made known in these columns **free of charge.**
6. All communications to be addressed to The Editor of The Hospital, 28 Southampton Street, Strand, London, W.C., and marked "Bureau of Information."

### Needs and Helpers.

### Home for Weak-Minded Patient.

Prudence.—It is impossible to procure accommodation for chronic mental patients at the very low fees you mention, viz., 10s. to 12s. 6d. The reason is that this class is well provided for in mental hospitals all over the country, where this is the ordinary fee for middle-class patients who can contribute towards maintenance. It is far safer to have your relative in an institution under public management and inspection than in a small establishment at very low fees, and, in our experience, the dread of unhappiness from being placed in a mental hospital is quite unfounded.

### Artificial Limb for Cripple.

H. C. B.—We have arranged for a medical officer to see the railway employee in whom you are interested, with a view to altering the construction of the wooden leg which does not fit, and have no doubt that a good surgical instrument maker will be able to make an improvement if properly directed. It is, however, a very common experience of cripples to procure at large cost an artificial limb which refuses to do its work. The weight is usually more than can be comfortably carried on a limb enfeebled by amputation, and the instances in which such aids can be habitually worn are comparatively rare.

### After-Care for Delicate Boy.

F. M. D.—The child you name, who is recovering from chronic nephritis, seems to be quite suitable for the Home referred to in the reply to Anne L. in The Hospital of August 10, p. 493, except that perhaps there might be a difficulty about supplying special diet. If, however, it is merely a case where ordinary wholesome food would suffice he would be eligible, and we will do our best to procure his admission. Will you kindly let us know about the diet and whether nursing is required? Also whether he is well enough to attend the village school, which is only across the road, under a very kind mistress.

### Training and Employment.

### Private Work in Italy.

N. M., Monkstown.—It is a little late for obtaining an engagement in Italy as the staff at institutes abroad is usually made up in the summer. Write, however, to the Anglo-American Nursing Home, 265 Via Nomentana, Rome, and to the Association of Trained Nurses, 7 Via Rondinelli, Florence. State fully and clearly your training and every kind of experience you have had. Enclose typed copies of testimonials and certificates and purchase a postal coupon to enclose for reply.

### Fever Hospital in Montreal.

D. A. M., Southsea.—The Hôpital Notre-Dame in Montreal has a branch establishment for the reception of contagious diseases at the "St. Paul's Hospital," 656 Maisonneuve Street, Montreal. It is nursed by sisters of charity, but a few pupil nurses are received. There are private rooms for paying patients and separate pavilions for diphtheria, scarlet fever, measles, etc. Apply to the Sister Superior, and mention previous training, if any.

### Training in Dublin.

Anne D., Southport.—Excellent training is to be had in Dublin, but premiums are expected. Apply to the matron of any of the following hospitals, and ask for a form of application to become probationer: Adelaide Hospital, premium £15; Dr. Steevens' Hospital, premium £54 12s.; House of Industry Hospitals, premium £50; Mater Misericordiæ Hospital, premium £40; Sir Patrick Dun's Hospital, premium £25. For information about American training schools see "How to Become a Nurse," The Scientific Press, Ltd., 28 Southampton Street, Strand, price 2s.

### Addresses of Nursing Homes in London.

H. E. A., Aberdeen.—Study the advertisements in the nursing papers, where Nursing Homes are constantly announcing their needs. Have your testimonials typed and enclose a copy with a clearly written and carefully thought-out letter in response to any vacancy likely to suit you. It would be useless to send you addresses, as the matrons might not be in need of nurses at the moment. You must watch your opportunity.

### Homes Wanted.

### At St. Leonards-on-Sea.

M. A. N. [116a], Cross-in-Hand.—As you are exposed to frequent attacks of illness we are sure you would be wise to seek accommodation at the sea, at any rate for part of the winter. We shall be happy to put you in communication with any Nursing Home on our List at St. Leonards-on-Sea which may be willing to receive you for a guinea and a half or two guineas a week.

### For Elderly Lady Feeble-Minded.

A. D. W. (117a).—We are pleased to make it known that you are looking for a Home for a lady aged seventy-four who suffers from loss of memory, and that her friends can pay £2 2s. weekly. We will forward replies from any Homes on our List which can receive such a case.

### Approved Homes.

The correct address for the private Home for nerve cases and after-care at Chelsfield, of which mention was made in our issue of August 3, is Mrs. Newman, Normanton, Chelsfield, Kent.

### Letter-Box.

K. C. A., Wimbledon.—Very glad to know of your offer *re* infants wanting a kind home.

H. H. D.—No financial help is needed at the moment. Should it be required no doubt a definite appeal will be made to the public, but it is well to be cautious.

M. C. White.—You cannot do better than study the advertisements in the paper you name, and answer such as appear suitable. Get the paper the day it comes out, answer promptly and persevere.

"E. D'O."—We have sent on your letter, and hope you may secure the post.

**The leaflet containing particulars of the Bureau is now ready and will be forwarded free on application.**

### Appointments.

Dr. Frederick Hall, Assistant Medical Officer of Health for Lincoln, has been appointed assistant school medical officer by the Derby Borough Education Committee.

Mr. Tyrell Gray, F.R.C.S., has been appointed surgeon to the West London Hospital. Mr. Gray, who is a graduate of Cambridge, has been surgeon to out-patients at the hospital.

Dr. C. M. Hinds Howell, M.D., F.R.C.P., has been appointed assistant physician to out-patients at the National Hospital, Queen Square. Dr. Howell has been pathologist and formerly registrar at the hospital.

The Hampstead Borough Council has appointed Dr. F. E. Scrase as the borough's medical officer of health to succeed Dr. G. F. McCleary, who has been appointed principal medical officer to the National Health Insurance Commission (England).

Dr. John Jekyll Rainforth, of Lincoln, has been appointed medical officer to the Lincoln General Dispensary. Dr. Rainforth, who took the conjoint diploma in 1904, graduated M.B. at London University, and took the F.R.C.S. in 1909. His previous appointments include several resident posts at the London Hospital.

Dr. A. E. Barnes has been appointed honorary physician to the Sheffield Royal Infirmary in succession to Dr. Dyson, who has resigned after twenty-seven years' service. Dr. Barnes has taken the degrees of M.B. at London and M.B., Ch.B. at Sheffield. He was formerly senior house physician and senior house surgeon at the Royal Infirmary.

Dr. Agnes Savill has been appointed hon. physician on the staff of the London Skin Hospital, Fitzroy Square, W. Dr. Savill is an M.D. of Glasgow University, and her previous appointments include those of physician to the Skin Hospital, Leicester Square, house surgeon to the Belgrave Hospital for Children, and assistant medical officer to the Toxteth Park Workhouse, Liverpool.

The following house appointments have been made at St. Thomas's Hospital from August 20, 1912 (subject to the approval of the Treasurer): To be casualty officers and resident anæsthetists: Mr. S. H. Rouquette, B.A. Cantab., M.R.C.S., L.R.C.P.; Mr. H. J. B. Fry, B.A., M.B., B.Ch., B.Sc. Oxon., M.R.C.S., L.R.C.P.; Mr. W. M. Oakden, B.A. Cantab., M.R.C.S., L.R.C.P.; Mr. W. D. Ross, B.A., M.B., B.C. Cantab., M.R.C.S., L.R.C.P.; Mr. G. N. Brandon, M.R.C.S., L.R.C.P.; Mr. W. G. Marsden, B.A., M.B., B.C. Cantab., M.R.C.S., L.R.C.P.; Mr. A. R. Chavasse, M.A., B.M., B.Ch. Oxon., M.R.C.S., L.R.C.P.; Mr. J. B. A. Wigmore, B.A., M.B., B.C. Cantab., M.R.C.S., L.R.C.P. To be house surgeon to Block 8: Mr. C. V. Anderson, M.R.C.S., L.R.C.P. To be obstetric house physicians: Mr. C. W. Treherne, M.R.C.S., L.R.C.P.; and Mr. G. E. Downs, M.B., B.Ch. Oxon. To be ophthalmic house surgeons: Mr. D. S. Bryan-Brown, B.A. Cantab., M.R.C.S., L.R.C.P.; and Mr. P. Verdon, B.A., M.R.C.S., L.R.C.P.

### ORDER OF ST. JOHN OF JERUSALEM.

The following among other promotions in, and appointments to, the Order of the Hospital of St. John of Jerusalem in England have been made: As Knight of Justice (from Knight of Grace): Harold Edwin Boulton, Esq., M.V.O. As Knights of Grace: John William Springthorpe, Esq., M.D. (from Honorary Associate); Lieutenant-Colonel Sir Joseph Fayrer, Baronet, F.R.C.S. Edin., M.D. (late R.A.M.C.); Major Charles Alfred Hodgetts, M.D., L.R.C.P., A.M.S. Canada (from Esquire). Sir Joseph Fayrer is, of course, Superintendent of the Edinburgh Royal Infirmary.

### Gifts and Bequests.

The Princess Henry of Battenberg has given £5 towards the rebuilding of the Nurses' Home of the Chelsea Hospital for Women.

Mrs. Louisa Grace Robertson-Aikman, of Albert Bridge Road, Chelsea, S.W., has left £1,000 to the Home for Incurables, Streatham.

The Queen has sent £10 10s. towards the rebuilding of the Chelsea Hospital for Women, and £10 10s. towards the rebuilding of its Nurses' Home.

Mr. Charles Janvrin Robin, of Jersey, barrister-at-law, has left £500 to the London Metropolitan Hospital Sunday Fund and £500 to the Jersey General Dispensary.

The Rev. Gordon Adam Stephenson, of Lee Terrace, Blackheath, S.E., and of Villa Gourdon, Cannes, France, who left £49,222, has bequeathed £500 to the Invalid Ladies' Home, Le Cannet, Cannes.

Mr. A. V. Pryor, D.L., J.P., has sent a donation of £52 10s. to the Leicester Royal Infirmary. Mr. Pryor has on many occasions contributed liberally to the funds of the institution, of the board of which he is a member.

An important anonymous gift was received by the West London Hospital on Saturday, when a cheque for £3,000 was received. The cheque bore two signatures, apparently on behalf of a third party, and the identity of the donor has so far been concealed.

The Hampstead General and North-West London Hospital has received an annual subscription of £50 from H.I.H. the Grand Duke Michael of Russia (president of the hospital), also a donation of £400 from "Anonymous," per Messrs. Robarts, Lubbock and Co.

The Hospital Saturday Fund (London) has received a bequest of £261 from the Executors of the late Miss Elizabeth Preedy, of Buckingham, and a gift of £100 Queensland Government Three per Cent. Inscribed Stock from the Proprietors of the Continental Daily Parcels Express—the interest to be distributed annually.

Miss Margaret Holland, of Uplyme, Devon, has bequeathed £100 each to Lyme Regis Cottage Hospital, Axminster Cottage Hospital, and Epsom Cottage Hospital, and £10,000 upon trust for her brother for life, with remainder to his daughter and her children or remoter issue, or, if none, then in equal shares to the above-named hospitals.

### Buildings Contemplated.

Dublin.—The Dublin Guardians will apply for sanction to a loan of £10,375 for nursing quarters at the Workhouse and alterations and additions at the hospitals and mental wards.

Manchester.—The Guardians of the Poor in the township of South Manchester invite tenders for new lunatic wards and other works at Withington Workhouse. Mr. F. H. Overmann, F.M.S.A., of Manchester, has prepared plans, and quantities may be obtained from him on deposit of one guinea.

Neath.—The Neath Guardians have received sanction to borrow £24,000 for a new infirmary.

Norwich.—The Guardians have approved plans prepared by the surveyor for a new block of children's homes at an estimated cost of £2,956.

Rawtenstall.—At a cost of over £30,000 the new infirmary in Haslingden Road has been completed and formally opened.

St. Austell.—The sanction of the Local Government Board is to be asked for a loan covering workhouse alterations and the erection of a mortuary, the estimated costs being £2,000 and £60 respectively.

Sandhurst, Berks.—The Secretary of State for War invites tenders for the erection of a twenty-bed hospital and other works at the Sandhurst Military College. Particulars may be had from the Director of Barrack Construction, 80 Pall Mall, S.W.

Somerset.—In connection with insurance legislation the Somerset County Council have decided to establish a 100-bed sanatorium in a central position, and also four main dispensaries at Taunton, Weston-super-Mare, Glastonbury, and Bath or Radstock.

South Africa.—The *South African Gazette* states that a contract has been entered into for the erection of a hospital at Maitland, Cape Province, at a cost of £18,031.

Stockport.—The result is announced of the architectural limited competition for additional mental wards at Stepping Hill Infirmary, Stockport. Messrs. E. T. and E. S. Hall, of London, are placed first; Mr. Arthur Marshall, of Nottingham, second; and Messrs. Spalding and Theakston, of London, third. The drawings have been on exhibition in Stockport for a short period.

Watford.—Mr. W. H. Syme, F.R.I.B.A., is architect for additions to Watford Union House for the Guardians. The works include additional boiler accommodation, a mortuary, and post-mortem room. Tenders are invited.

Willesden.—For the erection of Children's Homes adjoining Acton Lane Receiving House ten tenders have been received on plans prepared by Mr. J. Cash, F.R.I.B.A. The lowest amounts to £7,773, the highest to £9,729.

Winchcomb.—Messrs. Phillpott and George, of Cheltenham, are architects for alterations and additions to Winchcomb Workhouse Infirmary. Tenders for building and heating works, amounting to £2,084 and £123 respectively, have been accepted, subject to the approval of the Local Government Board.

Winchester.—An inquiry is to be held by the Local Government Board into application for sanctions to loans in respect to the erection of a joint mental hospital near Basingstoke, at Park Prewett Farm. The Southampton County Council are responsible for £171,009, the Town Council for £136,808, and the Bournemouth Town Council for £49,600.

### The Week's Novelties.

### ANTIDIPHTHERITIC SERUMS.

Upon the value of antidiphtheritic serum it is superfluous at the present day to dwell; but it is still necessary to insist that for use in conditions of so critical a character the serum must be absolutely reliable in purity and potency. Those medical men who have had the opportunity of seeing the conditions under which Messrs. Parke, Davis and Co. prepare their serum have no fear on this score. The elaborate precautions taken throughout all the stages, the care with which their animals are treated, and the meticulous accuracy with which the doses are measured cannot but inspire confidence in the users of the serum—a confidence which is, moreover, justified by the results obtained in actual treatment of diphtheria cases. The use of concentrated serums—that is, serums which contain from two to three times the number of antitoxin units per cubic centimetre which the "ordinary" antitoxin contains—is steadily increasing. The advantages of this are manifest, especially in dealing with severe cases in children. Messrs. Parke, Davis and Co. supply both a "standard" serum and also a "special" serum much more concentrated, which not only ensures a more prompt effect but very greatly diminishes the likelihood of the appearance of antitoxin rashes and joint-pains.

### AUTUMN PLEASURE CRUISES.

From London to Dalmatia and Venice by the *Vectis* on September 6, and to Lisbon, Teneriffe, Madeira, and Gibraltar by the *Mantua* on September 12, are the autumn cruises announced by the P. and O. Company. Besides Corfu and Venice, the former cruise includes a new port of call, Klek Bay, whence an excursion will be made to Mostar, the Herzegovinian capital; and with respect to the latter two days will be spent at each of the four ports to be called at. Illustrated handbooks of these cruises are obtainable at the Company's offices.

### Books Received.

T. Fisher Unwin: "Suggestion and Psychotherapy," by G. W. Jacoby, M.D.

W. B. Saunders Company: "Home Nurses' Handbook," by C. Aikens.

C. Knight and Co.: "Knight's Handy Insurance Cabinet."

W. H. and L. Collingridge: "The City of London Year Book and Civic Directory, 1912."

Headley Bros.: "The Problem of Consumption," by a Birmingham Physician.

H. K. Lewis: "Sea-Sickness and Health," by Joseph Byrne; "The Extra Pharmacopœia," by Martindale and Westcott, 2 vols.; "Elements of Practical Medicine," by H. H. Carter, M.D.

Burlington House, Cambridge: "Matriculation Directory, June 1912."

The Scientific Press, Ltd.: "Hospital Sisters and their Duties," by Eva Lückes, Matron, London Hospital.

Adam and Charles Black: "Harrogate," Black's Guide-Book series.

Chas. H. Sell: "Low's Handbook to the Charities," London, 1912.

Constable and Co.: "The Task of Social Hygiene," by Havelock Ellis.

J. Murray: "Tuberculosis and the Immune Substances (I.K.) Therapy," by W. H. Fearis.

Medical Review of Reviews: "An Essay on Hasheesh," by Victor Robinson.

John Wright and Sons: "Statistics of Puerperal Fever," by G. Geddes, M.D.

